# Baby‐Friendly Hospital designation has a sustained impact on continued breastfeeding

**DOI:** 10.1111/mcn.12497

**Published:** 2017-08-10

**Authors:** Anna Spaeth, Elisabeth Zemp, Sonja Merten, Julia Dratva

**Affiliations:** ^1^ Swiss Tropical and Public Health Institute Basel Switzerland; ^2^ University of Basel Basel Switzerland; ^3^ ZHAW University of Applied Sciences, School of Health Professions Institute of Health Sciences Zurich Switzerland

**Keywords:** Baby‐Friendly Hospital, breastfeeding, continued breastfeeding, exclusive breastfeeding, sustainability, *Ten Steps*

## Abstract

The Baby‐Friendly Hospital (BFH) Initiative has led to an increase in breastfeeding rates and duration worldwide. But little is known about whether the beneficial effects persist beyond a facility's designation as a BFH. To investigate the association of BFH designation (current, former, and never) and compliance with Baby‐Friendly (BF) practices on breastfeeding in Switzerland, this study combined nationwide survey data on breastfeeding with BFH monitoring data. In this cross‐sectional study, 1,326 children were born in 34 current (*N* = 508), 28 former (*N* = 425), and 34 never designated BFHs (*N* = 393). We compared exclusive and any breastfeeding according to BFH designation over the first year of life, using Kaplan‐Meyer Survival curves. Logistic regression models were applied to analyse breastfeeding prevalence, and Cox‐regression models were used for exclusive (0–6 months) and continued (6–12 months) breastfeeding duration. Average duration of exclusive breastfeeding (13.1 weeks, 95% confidence interval [12.0, 17.4]) and any breastfeeding (32.7 weeks, 95% confidence interval [30.5, 39.2]) were the longest for babies born in currently accredited BFHs. Exclusive breastfeeding was associated with high compliance with monitored BF practices in current BFHs and with the number of BF practices experienced in all hospitals. Continued breastfeeding was significantly longer when babies were born in current BFHs (cessation hazard ratio 0.60, 95% confidence interval [0.42, 0.84]) or in former BFHs (cessation hazard ratio 0.68, 95% confidence interval [0.48, 0.97]). Overall, the results support continued investment into BFHs, because babies born in current BFHs are breastfed the most and the longest, whereas a former BFH designation shows a sustained effect on continued breastfeeding.

## 1 INTRODUCTION

The Baby‐Friendly Hospital Initiative (BFHI) launched by WHO/UNICEF has proven to be a powerful tool for raising breastfeeding rates. Breastfeeding is a protective factor for health (Victora et al., 2016); thus, breastfeeding promotion continues to be an important measure to improve child and maternal health in both developed and developing countries. According to recent meta‐analyses, the BFHI was the most effective intervention for improving breastfeeding rates at health system level (Sinha et al., 2015) and adherence to the BFHI *Ten Steps* to support successful breastfeeding had a positive impact on breastfeeding outcomes (Perez‐Escamilla, Martinez, & Segura‐Perez, 2016).

In industrialised countries, 9% of facilities have been designated as a “Baby‐Friendly Hospital” (BFH) at least once. In a global context, this rate is low, but there are large variations in the proportion of BFHs across industrialised countries (Labbok, 2012). For example, in Sweden and The Netherlands, most hospitals have been designated as a BFH (97% and 83%, respectively), and in the United States and Canada, BFH designation rates are much lower (4% and 12%, respectively) (Labbok, 2012). In Switzerland, the BFHI has been rather successful, with 55% of hospitals having ever been designated a BFH in 2005 (Labbok, 2012). However, in 2013/2014, the proportion of designated BFHs fell to 28%, with approximately one third (2013: 38%, 2014: 33%) of all deliveries taking place in a BFH in Switzerland (Spaeth & Zemp Stutz, 2014; Spaeth & Zemp Stutz, 2015). Even accounting for hospitals withdrawing from or losing the BFH certification, the overall number of BFHs increased up to 2005 (Forrester‐Knauss, Merten, Weiss, Ackermann‐Liebrich, & Stutz, 2013). Thereafter, BFHs decreased slightly and then markedly from 2012 onwards, when a hospital financing system based on Diagnosis Related Groups was introduced in Switzerland (Wild, Pfister, & Biller‐Andorno, 2012). Budgetary pressures accompanied the new financing system, and a conflict arose between financial objectives and time spent monitoring Baby‐Friendly (BF) practices and offering educational and emotional support to mothers (Conzelmann‐Auer, 2009; Furrer, Schwab, & Oetterli, 2005). These issues, combined with insufficient marketing of the BFH label (Furrer et al., 2005), led some hospitals to withdraw from the initiative.

The reduction of designated BFHs does not seem to have had an immediate effect on national breast feeding rates, as evidenced by the 2014 nationwide survey of infant feeding practices (Dratva, Gross, Spaeth, & Zemp, 2014). The 2014 survey yielded a median duration of 12 weeks for exclusive breastfeeding and 31 weeks for any breastfeeding, similar to the durations observed in the previous national survey in 2003 (Dratva et al., 2014). In 2003, Merten et al. showed that compliance with BF practices was highly associated with breastfeeding duration (Merten, Dratva, & Ackermann‐Liebrich, 2005). We combined data from the 2014 national survey of infant feeding practices with the BFH monitoring data. We hypothesised that BFH accreditation in the past, as indicated by a former BFH designation, had a sustained impact on national breastfeeding rates and duration and that breastfeeding success remains particularly high when BFHs comply closely with monitored BF practices.

**Key messages**

- Babies are breastfed the longest when they are born in currently designated Baby‐Friendly Hospitals and achieve high compliance with monitored Baby‐Friendly practices.
- The Baby‐Friendly Hospital designation may have a sustained effect on continued breastfeeding, as babies born in former BFHs were breastfed longer than babies born in never accredited hospitals.
- The number of Baby‐Friendly practices experienced is positively associated with exclusive breastfeeding duration.

## 2 METHODS

**Figure A1:**
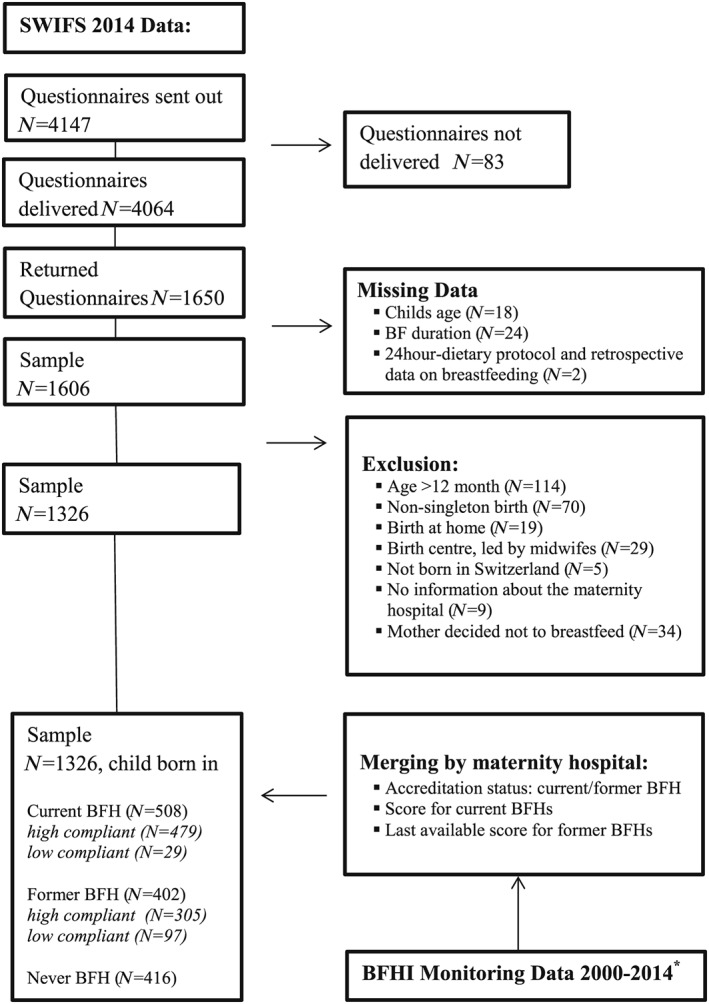
Flow‐chart of the study population

The Swiss Infant Feeding Study (SWIFS) is a nationwide cross‐sectional study on infant feeding practices and selected mother and child health outcomes during pregnancy and in the first year after birth (Dratva et al., 2014). A sample of mother–baby dyads was randomly selected (*N* = 4147) by Swiss Parent Counsellors from a list of births registered in the previous 11 months. The regional Parent Counselling Services (nationwide coverage with 158 services) routinely receive birth registry data from their respective communities. According to the study protocol, randomly selected mothers were sent a postal questionnaire and a reminder 2 weeks later. A total of 1,650 mothers responded, yielding a response rate of 40%. We excluded questionnaires due to missing data (*N* = 53), age (age > 12 months *N* = 114), nonsingleton birth (*N* = 70), place of birth (not born in a Swiss maternity hospital *N* = 53), and if the mother had decided not to breastfeed (*N* = 34). The remaining 1,326 mother–child pair data from the SWIFS study were merged with BFH monitoring (Fig. A1). The BFH monitoring data include information on monitored compliance with BF practices and the list of all designated BFHs (between the years 2000–2014). We defined hospitals as a current BFH if the health facility was a designated BFH in the year of birth; as a former BFH if the health facility was a designated BFH once before but not in the year of birth; and as a never BFH if the maternity ward had never been designated “Baby‐Friendly.”

### 2.1 Maternal, infant, and hospital characteristics

From the SWIFS survey, we obtained the following sociodemographic characteristics: maternal age, marital status, parental education (grouped according to tertiary education: none of the parents, one or both), linguistic region (German, French, or Italian speaking), nationality (Swiss or non‐Swiss), monthly household income (<6,000 CHF; 6,000–9,000 CHF; >9,000 CHF), current intake of hormonal contraception as reported by mother, smoking status, weight and height, age of the child when mother took up work, parity (first child or not), birth weight, gestational age, and mode of delivery. Age of the child when mother took up work was available in months or weeks and was categorised into three time periods: “<5 months,” corresponding to the paid maternity leave in Switzerland required by law; “5–6 months,” according to the WHO recommendation to exclusively breastfeed up to 6 months; and “>6 months.”

Hospitals were categorised according to their status as a teaching hospital, size, and ownership: “A‐level” corresponded to university or central teaching hospitals with a 4‐year postgraduate medical training, “B‐level” corresponded to regional hospitals with a 3‐year postgraduate medical training, “private hospital,” and “regional hospital.”

### 2.2 Compliance with Baby‐Friendly practices

To be accredited as a BFH, hospitals have to implement the *Ten Steps* to Successful Breastfeeding and adhere to the Code of Marketing of Breast‐milk Substitutes (WHO/UNICEF, 2009). Once accredited, BFHs are assessed every 3 to 5 years with an audit and with continuous monitoring of four of the *Ten Steps* (Steps 4, 6, 7, and 9; Forrester‐Knauss et al., 2013). Monitoring data are collected routinely by nurses or midwives on the maternity ward. Monitoring data are analysed annually, and a compliance score is calculated for each hospital (Table A1). For former BFHs, we merged the last available monitored compliance score; for current BFH, we used the score achieved in the year of birth. Based on a former study of BFH and compliance with monitored BF practices in Switzerland (Merten et al., 2005), we defined hospitals as “low compliant” if their score was <3 and as “high compliant” if their score was ≥3 on a scale ranging from 0 to 4.

Participating mothers provided information on the following steps: first attempt to breastfeed within 1 hr of birth (Step 4), receiving advice on how to breastfeed (Step 5), giving no food or drink other than breast milk (Step 6), rooming‐in for 24‐hours (Step 7), breastfeeding on demand (Step 8) and no pacifier use (Step 9). We defined these BF practices experienced and reported by the mothers as “reported compliance”, ranging from 0 to 6.

### 2.3 Breastfeeding

We assessed exclusive and any breastfeeding (see definition in Table A2) based on a 24‐hr dietary protocol as well as on retrospective reporting in the SWIFS study. Information about breastfeeding and first food or liquid was obtained from the questionnaire. Mothers were asked at what child age (in months or weeks) they had stopped exclusive or any breastfeeding, and when they introduced complementary food, water, and formula. Outcome variables were the duration of exclusive, any, and continued breastfeeding (Table A2).

### 2.4 Statistical analyses

Characteristics are presented as percentages and compared across BFH designations by using logistic regression models with random intercepts for hospitals. The associations of BFH designation with duration of exclusive and any breastfeeding, respectively, are displayed as Kaplan‐Meyer curves. Differences across BFH designations were assessed by using the Log‐Rank Test and by reporting median values with their 95% confidence intervals.

The prevalence of exclusive breastfeeding at 3 months and of continued breastfeeding at 6 and 9 months according to BFH designation was analysed by using mixed logistic regression models with random intercepts for hospitals, adjusting for covariates selected if *p* < .2 after backward selection (see footnotes in Table A5). In a sensitivity analyses, time since becoming a former BFH was included in the model.

To assess the association of BFH designation on exclusive breastfeeding and continued breastfeeding, we ran multivariable Cox‐regression models. The model was adjusted for covariates according to the literature and selected if *p* < .2 after backward selection (See footnotes, Table 3). For exclusive breastfeeding, data were censored if the child was exclusively breastfed and less than 6 months old. For continued breastfeeding, only children who were breastfed beyond 6 months were included and data were censored at the age of the child if the child was still being breastfed. As the proportional hazard assumption was violated for several covariates, we split the follow‐up time into periods of 1 month and added interaction terms between periods and the respective covariates. For exclusive breastfeeding, these covariates were education and parity; for continued breastfeeding, it was smoking status. We also introduced a time‐dependent indicator variable for “work having been resumed before the respective period.”

Cox‐regression was used to analyse the effect of monitored and reported compliance, as well as hospital characteristics on exclusive and continued breastfeeding in each group (current, former, and never BFH). Data were analysed using STATA (StataCorp LP, Texas, USA, version 14).

## 3 RESULTS

In our study, 508 children (38%) were born in 34 current, 425 (32%) in 28 former, and 393 (30%) in 34 never BFHs (Figure A1). In our study population, 70% of the children were older than 6 months and the mean age of the children was 7.5 months. Compared to all women who had given birth in Switzerland in 2013, SWIFS mothers were 1.7 years older and had a higher rate of primipara and a lower rate of caesarean sections (Table 1). When the study population was compared across BFH designations, characteristics were not significantly different (data not shown).

**Table 1:** Sample characteristics of mothers who intended to breastfeed and gave birth in a Swiss maternity hospital, and their children (*N* = 1,326)

Characteristic	Study population	Swiss population
Swiss nationality	76.8%	72%^a^
Income		
<6000 SFr	39.5%	–
6000–9000 SFr	31.9%	–
>9000 SFr	25.7%	–
Parental education		
No parent with tertiary education	28.5%	–
One parent with tertiary education	30.0%	–
Both parents with tertiary education	40.2%	–
German‐speaking region	76.3%	70.4%^a^
First child	54.1%	48.4%^a^
Caesarean section	30.4%	33.3%^b^
Birth weight 2500–4500 g	92.9%	92.7%^a^
Hormonal contraception	29.7%	–
Smoking	10.0%	–
Mother's return to work		
At child's age <5 months	22.0%	–
5–6 months	27.1%	–
>6 months	20.2%	–
No employment	30.6%	–
Median maternal age	32.8 years	31.6 years^a^
Median BMI	23.3	–
Birth in a current BFH	38%	37%^c^

a Birth registry 2013.

b Swiss Hospital Medical Statistics 2013.

c Yearly report on Baby‐Friendly hospitals 2013.

Hospital characteristics across BFH designations are shown in Table 2. High compliance with monitored BF practices was equally distributed among mother–baby dyads in current (86.6%) and former (86.4%) BFHs. Reported compliance showed similar results. Mothers reported having experienced 4.4 steps on average in current and former BFHs and 3.9 steps on average in never BFHs (data not shown). As shown in Table 2, two BF practices were significantly more frequent in current and former BFHs: exclusive breastfeeding during hospital stay (Step 6) and no pacifier use while in hospital (Step 9). Sensitivity analyses with data restricted to babies born at term with normal birth weight showed the same significant differences. The number of births in a private hospital was highest among never BFHs (Table 2). The median duration of BFH accreditation was 11 years in current and 9 years in former BFH (Tables A3 and A4). In the group of former BFHs (*N* = 27), time since becoming a former BFH varied between less than 1 year and up to 11 years (Table A4), with a median of 2 years.

**Table 2:** Hospital characteristics according to Baby‐Friendly Hospital designation

	Current (*N* = 508) %	Former (*N* = 425) %	Never (*N* = 393) %	Total (*N* = 1,326) %
Monitored compliance^a^				
Low	86.6	86.4	n.a.	86.0
High	13.4	13.6	n.a.	14.0
Reported compliance^b^				
Step 4	65.2	59.5	58.8	61.5
Step 5	82.1	82.8	84	82.9
Step 6	58.9*	62.4*	45.3	56.0
Step 7	70.9	71.3	63.6	68.9
Step 8	78.5	74.8	76.8	76.8
Step 9	70.3*	72.7*	40.5	62.2
Mother–child dyads in				
University or central teaching hospital (A‐level)	33.5	29.9	21.1	28.7
Regional teaching hospital (B‐level)	55.5	45.2	38.7	47.2
Private hospital	9.4	24.9	32.1	21.1
Other hospital	1.6	0	8.1	3.0

a Compliance with monitored Baby‐Friendly practices is calculated annually, based on continuous data collection on four of the *Ten Steps* for Successful Breastfeeding (Steps 4, 6, 7, and 9) in Baby‐Friendly Hospitals (BFH). We defined hospitals as “low compliant” if their score was <3, and as “high compliant” if their score was ≥3 (range 0–4). For babies born in former BFHs, we merged the last available monitored compliance score and for those born in current BFHs, the score achieved in the year of birth.

b Mother reported on skin to skin contact immediately after birth with first attempt of breastfeeding within 1 hr after birth (Step 4), getting advice on breastfeeding during hospital stay (Step 5), giving the baby no food or drink other than breast milk (Step 6), rooming‐in for 24 hr (Step 7), breastfeeding on demand (Step 8), and no use of pacifiers (Step 9).

* Logistic regression models with random intercepts for hospitals compared to never BFH: *p* < .05.

**Figure 1:**
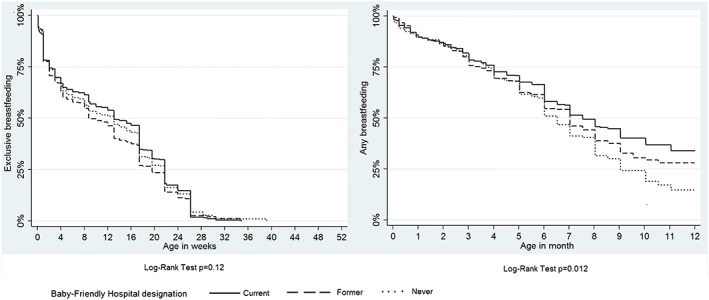
Kaplan‐Meyer analyses of exclusive and any breastfeeding according to Baby‐Friendly Hospital designation

Kaplan‐Meyer curves for exclusive breastfeeding (Figure 1) showed the most prominent differences according to BFH designation up to week 17, when most of the babies (98%) had not yet been introduced to complementary food. The median duration of exclusive breastfeeding was 13.1 weeks in current BFHs (95% confidence interval [12.0, 17.4]), 8.7 weeks in former BFHs (95% confidence interval [8.0, 13.1]), and 13.1 weeks in never BFHs (95% confidence interval [8.7, 15.2]).

Kaplan‐Meyer curves for any breastfeeding appear to diverge after around 6 months of life (Figure 1). Between current and former versus never BFHs, our best fitting model described the natural logarithms of hazard ratios as linear functions of time supporting a steady decrease in hazard ratios over time. The median duration of any breastfeeding was 32.7 weeks in current BFHs (95% confidence interval [30.5, 39.2]), followed by 30.5 weeks in former BFHs (95% confidence interval [26.1, 32.7]) and 28.3 weeks in never BFHs (95% confidence interval [26.1, 30.5]).

The rate of exclusively breastfed babies was highest for babies born in current BFHs (Table A5). While the unadjusted rates showed significant differences in exclusive breastfeeding between current (51.7%) and former BFHs (43.0%) at 3 months, adjusted rates showed no significant difference according to BFH designation. The number of exclusively breastfed children at 6 months of age in our study population was very low (3%). The rate of continued breastfeeding at 6 and 9 months (Table A5) supported our model of the growing gap in breastfeeding rates between current, former, and never BFH from 6 months onwards. In our study population, only 98 children were aged 12 months, of whom 24.5% were still breastfed.

As shown in Table 3, babies born in current or former BFHs were breastfed significantly longer compared to those born in never BFHs. The adjusted cessation hazard ratio for babies born in current BFHs was 0.60 (95% confidence interval [0.42, 0.84]) and in former BFHs 0.68 (95% confidence interval [0.48, 0.97]).

**Table 3:** Cessation of exclusive and continued breastfeeding according to Baby‐Friendly Hospital (BFH) designation

	Cessation of exclusive breastfeeding (*N* = 1326)		Cessation of continued breastfeeding (*N* = 510)	
BFH designation	Adjusted HR^a^	95% CI	Adjusted HR^b^	95% CI
Never	1		1	
Former	1.11	0.94–1.32	0.68	0.48–0.97
Current	0.99	0.84–1.16	0.60	0.42–0.84

*Note*. Duration of exclusive breastfeeding was censored after 6 months, and duration of continued breastfeeding was censored before 6 months. HR = hazard ratio.

a Adjusted for mother's age, parental income (<6,000 CHF; 6,000–9,000 CHF; >9,000 CHF) and education (no parent with tertiary education, one with tertiary education, both with tertiary education), linguistic region (German, yes/no), parity (first child or not), age of infant when mother took up work again (in weeks), and mother's smoking status.

b Adjusted for mother's age and Swiss nationality, parental income (<6,000 CHF; 6,000–9,000 CHF; >9,000 CHF) and education (no parent with tertiary education, one with tertiary education, both with tertiary education), age of infant when mother took up work again (in weeks), mother's smoking status, and current intake of hormonal contraception.

We found that low compliance with BF practices was a strong predictor of shortened duration of exclusive breastfeeding (Table 4). Monitored high compliance was positively associated with exclusive breastfeeding in current BFHs (cessation hazard ratio 0.62, 95% confidence interval [0.42, 0.91]). Reported compliance, expressed as the number of experienced BF practices (where higher compliance corresponds to a greater number of experienced BF practices), was positively associated with exclusive breastfeeding duration in current, former, and never BFHs (Table 4). While we observed no significant association for continued breastfeeding with hospital characteristics in current or former BFHs, babies born in a university and central teaching hospitals (A‐level, Reference) of the never BFH group had a significantly lower risk of stopping continued breastfeeding compared to regional teaching (B‐level) hospitals (cessation hazard ratio 2.20, 95% confidence interval [1.14, 4.26]).

**Table 4:** Cessation of exclusive and continued breastfeeding according to hospitals' characteristics in current, former, and never Baby‐Friendly Hospital (BFH)

	Cessation hazard ratio for exclusive breastfeeding in									Cessation hazard ratio for continued breastfeeding in								
Characteristic	*N*	Current		*N*	Former		*N*	Never		*N*	Current		*N*	Former		*N*	Never	
Monitored compliance high	508	0.62	(0.42–0.91)	425	0.93	(0.71–1.22)		–		213	0.56	(0.24–1.28)	160	0.80	(0.45–1.41)		–	
Number of Baby‐Friendly practices experienced	459	0.85	(0.77–0.92)	375	0.81	(0.74–0.89)	338	0.89	(0.81–0.97)	199	0.97	(0.80–1.19)	143	0.90	(0.74–1.09)	127	1.06	(0.87–1.29)
Time since becoming former BFH		–		425	1.02	(0.99–1.06)		–			–		160	0.98	(0.91–1.05)		–	
Hospital characteristics^a^	508			425			393			213			160			137		
B‐level		0.94	(0.75–1.17)		1.08	(0.84–1.39)		1.09	(0.80–1.49)		1.22	(0.73–2.05)		1.41	(0.80–2.49)		2.20	(1.14–4.26)
Private		0.98	(0.68–1.43)		1.15	(0.85–1.54)		1.09	(0.79–1.51)		0.60	(0.23–1.60)		1.29	(0.67–2.49)		1.38	(0.66–2.88)
Regional		1.37	(0.60–3.12)		–	–		1.09	(0.66–1.80)		0.51	(0.07–3.78)		–	–		2.36	(0.88–6.32)

A‐level hospitals (Reference) are teaching hospitals with a 4‐year postgraduate medical training; these are university hospitals or large central hospitals (1,300–2,500 births per year). B‐level hospitals are teaching hospitals with a 3‐year postgraduate medical training; these are middle‐size hospitals (300–1,500 births per year).

## 4 DISCUSSION

The results of our study showed that BFH designation was associated with continued breastfeeding but not with exclusive breastfeeding duration. However, exclusive breastfeeding duration was associated with mothers' reported compliance to six of the *Ten Steps* in any hospital and with high compliance to monitored BF practices in current BFHs. Our results also demonstrate that mother–baby dyads from former BFHs had higher rates of continued breastfeeding than those from never BFHs, which supports our hypothesis of a partial sustainability of BFH accreditation.

### 4.1 BFH designation and continued breastfeeding

For continued breastfeeding from 6 months onwards, the likelihood of receiving mother's milk was higher for babies born both in current and former BFHs. Although the association with current BFHs was expected, the effect in former BFHs is new and indicates a partial sustainability of the BFH designation. In 2001, a randomised trial in Belarus yielded significantly higher rates of any breastfeeding at 12 months when the baby had been born in a current BFH (Kramer et al., 2001); in Turkey, breastfeeding rates and prolonged breastfeeding lasting more than 12 months increased after BFH accreditation of a university hospital (Duyan Camurdan et al., 2007). These two studies indicate that the process of accreditation has a positive impact on continued breastfeeding. A limitation of this study is that we lack information from current and former BFHs on Steps 1, 2, 3 and 10. However, although these steps are implemented in current BFHs and reassessed every 3 years, former BFHs would have had a written breastfeeding policy and healthcare staff trained in skills necessary to implement this policy. We hypothesise that the sustained effect observed is related to the accreditation process. The time spent on training staff, strengthening a positive and encouraging attitude towards breastfeeding at a hospital during the accreditation process, and promoting breastfeeding using the *Ten Steps* to Successful Breastfeeding is not lost immediately when a hospital steps out of the BFHI.

Lower breastfeeding rates in hospitals never designated as a BFH, compared to current and former BFHs, might also relate to different experiences after discharge. Community support (Step 10) appears to be essential for sustaining the breastfeeding impact of the BFH (Perez‐Escamilla et al., 2016). Current and former BFHs would have implemented Step 10 as part of the accreditation process. As we lack information on Step 10, we have no information on its implementation in the group of never BFHs. However, as private hospitals were overrepresented in the group of never BFH, one could assume that these private hospitals have a weaker link to community‐based breastfeeding support.

### 4.2 Compliance with Baby‐Friendly practices and excusive breastfeeding

The number of BF practices experienced and reported by the mother was positively associated with exclusive breastfeeding. Previous studies have shown that the number of Baby‐Friendly practices experienced has a positive effect on short‐term breastfeeding, irrespective of BFH designation (Brodribb, Kruske, & Miller, 2013; Callendret et al., 2015; Chien, Tai, Chu, Ko, & Chiu, 2007; Dulon, Kersting, & Bender, 2003; Murray, Ricketts, & Dellaport, 2007). Although our data support this view, they also point to the necessity of high compliance with monitored compliance, as previously shown in studies in Switzerland (Merten & Ackermann‐Liebrich, 2004; Merten et al., 2005). A high level of monitored compliance proved to be strongly associated with exclusive breastfeeding duration and positively associated with continued breastfeeding.

We could show that Step 6 (exclusive breastfeeding during hospital stay) and Step 9 (no pacifier use) were significantly more often experienced both in current and former BFHs. It was shown that Step 6, in particular, may help mothers to achieve their goal of breastfeeding exclusively (Perrine, Scanlon, Li, Odom, & Grummer‐Strawn, 2012). Therefore, it was rather surprising that exclusive breastfeeding rates and duration did not differ significantly according to BFH designation. It seems that compliance and practicing the steps are more important than designation to enable women to breastfeed exclusively. As BF practices were reported by the mother as experienced or not, it may reflect their breastfeeding self‐efficacy. Breast feeding self‐efficacy is an important independent predictor for breastfeeding duration (Baghurst et al., 2007; Scott, Shaker, & Reid, 2004). The reported experience could therefore be biased by mothers' self‐efficacy, which we did not account for by using the Iowa Infant Feeding Attitude Scale (De la Mora & Russell, 1999). One strength of the study is that we used two sources of information about BF practices as recommended (Haiek, 2012); monitored high compliance showed the same effects on breastfeeding exclusivity as reported compliance. Given that all mothers in our study population intended to breastfeed, we hypothesise that BF practices positively influence breast feeding self‐efficacy and vice versa.

### 4.3 Strengths and limitations

A major limitation of this study is the lack of information on nonrespondents (60%). Comparing to all mothers, our study sample is overrepresented by older Swiss women living in the German speaking part of Switzerland, with their first child. In the United States, the impact of BFHI has been higher among mothers with lower education levels (Hawkins, Stern, Baum, & Gillman, 2014). The overrepresentation of highly educated parents would, therefore, likely lead to an underestimation of the effect, as characteristics of the study population were not significantly different across BFH designations.

To minimise recall bias, we excluded children >12 months. The high rate of children older than 6 months at the time mothers filled in the questionnaire may nevertheless have introduced some recall bias for exclusive breastfeeding and the reported compliance with BF practices. However, we do not expect a differential misclassification because “Baby‐Friendly” was not mentioned at any point in the study.

One might argue that maternal choice of hospital might have biased the results. However, for our study, we excluded mothers who, already before having given birth, had no intention of breastfeeding their child. Therefore, we do not think that the results are biased by mothers' intention to breastfeed. Furthermore, in Switzerland, the decision of where to give birth depends on the place of residence, the health insurance or the gynaecologist (in case of privately insured mothers) rather than on the Baby‐Friendly label (Furrer et al., 2005).

Although we have information on reported BF practices for all hospitals, monitored compliance is not available for never BFHs and outdated for former BFHs. But given the high rate of former BFHs in (high) compliance with monitored BF practices at the time of giving up the label, we do not believe that quality issues were the main reason for stepping out of the BFHI.

The linkage between the detailed SWIFS data and the BFHI monitoring data enabled us to find some preliminary answers to questions about the sustainability of BFH accreditation benefits and confirm the importance of BFH designation and compliance with the *Ten Steps* to Successful Breastfeeding. Our data were, however, limited to monitoring compliance with four steps and mothers' reports on whether or not they experienced Steps 4 to 9. These quantitative methods may not be sufficient to explain the sustained effect of BFH accreditation. Future research should apply a mixed‐methods approach to better discover how the *Ten Steps* are implemented across hospitals.

## 5 CONCLUSIONS

This study showed that outcomes for exclusive breastfeeding and continued breastfeeding were different. Although BF practices had a positive impact on exclusive breastfeeding, BFH designation had a positive effect on continued breastfeeding. It is important to avoid falling into the trap of asking the wrong question, namely, which is more relevant to breastfeeding outcomes. Besides healthcare services, social attitudes and values, and women's work and employment conditions need to be addressed to enable women to breastfeed (Rollins et al., 2016). In the study population, partners who explicitly encouraged mothers to breastfeed had a positive effect on exclusive breastfeeding duration compared to partners who were open to mixed feeding (Dratva et al., 2014). Mothers' work negatively influenced exclusive breastfeeding duration, as did living in the French speaking part of Switzerland (Dratva et al., 2014). We hypothesise that, in the Swiss context, BF practices only have a short‐term impact on exclusive breastfeeding outcomes, as it is impaired by factors like work, social attitudes, and values. In former BFHs, we observed a positive association with continued breastfeeding similar to that of current BFHs—an encouraging finding. This suggests that past training efforts and implementation of the *Ten Steps* to Successful Breastfeeding have had a sustained effect on breastfeeding practice and duration. The discrepant findings between exclusive breastfeeding associated with BF practices and continued breastfeeding with BFH designation revealed that both initiatives are needed in combination. Reinforcing the accreditation of hospitals as Baby‐Friendly and investing in compliance are the best ways to reach and maintain high prevalence and long duration of breastfeeding.

## CONTRIBUTIONS

AS, MPH conceptualised and designed the paper, carried out the analyses, drafted the initial manuscript, and is the first author of the final manuscript as submitted. EZ designed the SWIFS study, participated in the interpretation and discussion of results reviewed, revised the manuscript, and approved the final manuscript as submitted. SM designed the monitoring of the BFH indicators, reviewed and revised the manuscript, and approved the final manuscript as submitted. JD designed the SWIFS study, led the SWIFS study data collection, participated in the interpretation and discussion of results, reviewed and revised the manuscript, and approved the final manuscript as submitted.

## CONFLICTS OF INTEREST

The authors declare that they have no conflicts of interest.

## 1

**Table A1:** Compliance score with achievement requirements for current BFH in Switzerland^a^

Score items	Monitored Baby‐Friendly practice^b^	Cut‐off	Score
Step 4a	Skin‐to‐skin contact within 1 hr after birth	80%	0.5
Step 4b	First suckling during first skin‐to‐skin contact within 2 hr after birth	80%	0.5
Step 6a	Fully breastfed at discharge	80%	0.5
Step 6b	Exclusively breastfed at discharge	50%	0.5
Step 7a	Permanent rooming‐in with allowance of 1 to 2 exceptions in between two meals	50%	0.5
Step 7b	At least one time with the mother for 24 hr	80%	0.5
Step 9a	No bottle feeding	80%	0.5
Step 9b	No pacifier use	66%	0.5

a The monitored step is achieved and given 0.5 point when the proportion of mother and child dyads fulfilling the step reaches the cut‐off point. Each step achieved adds up to a compliance score with a maximum of 4 points.

b Mother and child dyads are included in the monitoring if gestational age is 37–42 weeks, birth weight 2,500–4,500 g, hospital stay >24 hr, mother and child are healthy, mother intended to breastfeed, and if there was no contraindication to breastfeeding.

**Table A2:** Breastfeeding definitions

Exclusive breastfeeding	The infant receives only breastmilk and no other liquids or solids, with the exception of drops or syrups consisting of vitamins, mineral supplements, or medicines.
Any breastfeeding	The child receives breastmilk. This definition may include exclusive breastfeeding.
Continued breastfeeding	Continued breastfeeding is defined as breastfeeding beyond the age of 6 months.

**Table A3:** Characteristics of current Baby‐Friendly Hospitals (BFHs)

	Hospital characteristics	Duration on‐label (years)	High monitored compliance^a^ (N)	Low monitored compliance^a^ (N)	Mother child dyads in the study (N)
1	B level	1	14		14
2	B level	5	19		19
3	B level	5	14		14
4	Regional	7	4		4
5	B level	8	16		16
6	B level	8	14		14
7	B level	8	2		2
8	B level	9	24		24
9	B level	9	22	1	23
10	B level	10	16		16
11	B level	10	10		10
12	A level	10	15		15
13	B level	10	2		2
14	B level	10	11		11
15	A level	10	36		36
16	B level	11	10		10
17	B level	11	22		22
18	B level	11	25		25
19	A level	11	39		39
20	Private	12	8		8
21	B level	12	4		4
22	B level	12	14		14
23	Private	12	11		11
24	B level	13	19		19
25	A level	14	36		36
26	Private	14	4	9	13
27	A level	16	2	19	21
28	Regional	16	2		2
29	B level	17	12		12
30	Private	17	16		16
31	B level	18	18		18
32	B level	19	9		9
33	Regional	19	2		2
34	B level	20	7		7
Median (years)		11			
Total (N)			479	29	508

*Note*. Hospital number 9 changed from high compliance in 2013 to low compliance in 2014. Hospitals numbers 26 and 27 changed from low compliance in 2013 to high compliance in 2014.

A‐level hospitals are teaching hospitals with a 4‐year postgraduate medical training; these are university hospitals or large central hospitals (1,300–2,500 births per year).

B‐level hospitals are teaching hospitals with a 3‐year postgraduate medical training; these are middle‐size hospitals (300–1,500 births per year).

a Compliance score from monitoring data at year of birth.

**Table A4:** Characteristics of former Baby‐Friendly Hospitals (BFHs)

	Hospital characteristics	Time since becoming a former BFH (years)	Duration on‐label (years)	High monitored compliance^a^ (N)	Low monitored compliance^a^ (N)	Mother child dyads in the study (N)
1	Private	<1	17	4		4
2	Private	<1	12	2		2
3	B level	1	15	4		4
4	B level	1	11	4		4
5	B level	1	10	4		4
6	B level	1	9	5		5
7	B level	1	4	26		26
8	Private	2	14	22		22
9	B level	2	13	22		22
10	B level	2	12	35		35
11	B level	2	10	18		18
12	B level	2	5	2		2
13	Private	2	9	8		8
14	B level	2	6	14		14
15	Private	3	9		18	18
16	A level	5	12	23		23
17	B level	5	9	7		7
18	A level	5	9	35		35
19	B level	6	11		18	18
20	B level	6	7		1	1
21	Private	7	5	15		15
22	Private	7	3		37	37
23	B level	9	2	23		23
24	B level	10	6		9	9
25	A level	11	2	38		38
26	A level	11	5	31		31
Median (years)		2	9			
Total (N)				342	83	425

*Note*. A‐level hospitals are teaching hospitals with a 4‐year postgraduate medical training; these are university hospitals or large central hospitals (1,300–2,500 births per year).

B‐level hospitals are teaching hospitals with a 3‐year postgraduate medical training; these are middle‐size hospitals (300–1,500 births per year).

a Compliance score from monitoring data at last year of having the label “Baby‐Friendly.”

**Table A5:** Prevalence of exclusive breastfeeding at 3 months and continued breastfeeding at 6 and 9 months according to Baby‐Friendly Hospital (BFH) designation

BFH designation	Exclusive breastfeeding at 3 months			Continued breastfeeding at 6 months			Continued breastfeeding at 9 months		
	N	%	Adjusted rate^a^ (95% CI)	N	%	Adjusted rate^b^ (95% CI)	N	%^+^	Adjusted rate^b^ (95% CI)
Never	170	48.7	51.2 (45.8–56.5)	178	66.4	67.8 (62.3–73.3)	61	41.5	42.0 (34.1–50.0)
Former	163	43.0	44.2 (39.0–49.4)	207	66.4	67.2 (62.0–72.4)	81	45.5	44.6 (37.0–52.3)
Current	241	51.7	52.2 (47.6–56.9)	265	69.9	69.1 (64.5–73.8)	105	49.8	50.6 (43.8–57.4)

a Covariates included in the model were adjusted for mother's age, parental education (no parent with tertiary education, one with tertiary education, both with tertiary education), mother's return to work (<5 months, 5–6 months, >6 months), parity (first child or not), caesarean section, hormonal contraception, and smoking status, for exclusive breastfeeding.

b Covariates included in the model were parental education (no parent with tertiary education, one with tertiary education, both with tertiary education), parental income (<6,000 CHF; 6,000–9,000 CHF; >9,000 CHF monthly), mother's return to work (<5 months, 5–6 months, >6 months), parity (first child or not), hormonal contraception, and smoking status, for continued breastfeeding.
